# Global, Regional and National Burden of Human Cystic Echinococcosis from 1990 to 2019: A Systematic Analysis for the Global Burden of Disease Study 2019

**DOI:** 10.3390/tropicalmed9040087

**Published:** 2024-04-17

**Authors:** Tian Tian, Liyuan Miao, Wei Wang, Xiaonong Zhou

**Affiliations:** 1 National Institute of Parasitic Diseases, Chinese Center for Disease Control and Prevention (Chinese Center for Tropical Diseases Research), WHO Collaborating Centre for Tropical Diseases, National Health Commission Key Laboratory of Parasite and Vector Biology, National Center for International Research on Tropical Diseases, National Key Laboratory of Intelligent Tracking and Forecasting for Infectious Diseases, Shanghai 200025, China; tiantian@nipd.chinacdc.cn; 2 School of Global Health, Chinese Center for Tropical Diseases Research, Shanghai Jiao Tong University School of Medicine, Shanghai 200025, China; miaoliyuan517@163.com; 3 One Health Center, Shanghai Jiao Tong University—The University of Edinburgh, Shanghai 200025, China; 4 National Health Commission Key Laboratory of Parasitic Disease Control and Prevention, Jiangsu Provincial Key Laboratory on Parasite and Vector Control Technology, Jiangsu Institute of Parasitic Diseases, Wuxi 214064, China; wangwei@jipd.com; 5 Hainan Center for Tropical Diseases Research, Haikou 571199, China

**Keywords:** cystic echinococcosis, burden of disease, disability-adjusted life year, age-standardized incidence rate, age-standardized mortality rate

## Abstract

Background: Cystic echinococcosis (CE) is a neglected tropical parasitic disease that poses huge disease, social and economic burdens worldwide; however, there has been little knowledge on the global morbidity, mortality and disability-adjusted life years (DALYs) of CE until now. This study aimed to collect the most up-to-date data about the global, regional and national disease burden due to CE from 1990 to 2019 and to project trends in the next 10 years. Methods: We measured the global, regional and national morbidity, mortality and DALYs of CE from 1990 to 2019 based on the Global Burden of Disease Study 2019 (GBD 2019) data, and we examined the correlation between socioeconomic development levels and the disease burden of CE. In addition, the disease burden due to CE was projected from 2020 to 2030. Results: The age-standardized incidence rate (ASIR) of CE reduced from 2.65/10^5^ [95% *UI*: (1.87/10^5^ to 3.7/10^5^)] in 1990 to 2.6/10^5^ [95% *UI*: (1.72/10^5^ to 3.79/10^5^)] in 2019 (EAPC = −0.18%). The number of deaths, DALYs, age-standardized mortality rate (ASMR) and age-standardized DALY rate due to CE all showed a tendency to decline from 1990 to 2019. A higher disease burden of CE was measured in women than in men in 2019. There was a significant difference in the ASMR of CE by region according to the socio-demographic index (SDI), and lower burdens of CE were estimated in high-SDI regions. The global ASIR of CE is projected to decline from 2020 to 2030; however, the ASMR and age-standardized DALY rate are projected to rise. Conclusions: The global burden of CE remains high, and it is recommended that more health resources are allocated to low-SDI regions, women and the elderly aged 55 to 65 years to reduce the disease burden of CE.

## 1. Background

Echinococcosis, caused by larvae of the genus *Echinococcus*, is a global, neglected, tropical zoonotic parasitic disease [1]. There are nine *Echinococcus* species that have been characterized worldwide until know, and there are four of concern in humans: *E. granulosus*, *E. multilocularis*, *E. oligarthrus* and *E. vogeli* [2]. *E. granulosus s.s.*, *E. ortleppi* and *E. canadensis*, which are part of the *E. granulosus s.l*. complex., cause cystic echinococcosis, and *E. multilocularis* cause alveolar echinococcosis, while *E. oligarthrus* and *E. vogeli* cause polycystic neotropical echinococcosis, which is limited to South America, Central America and North America [3].

As a neglected tropical disease, cystic echinococcosis is most prevalent in impoverished rural communities where animal husbandry is common [1]. Patients suffering from cystic echinococcosis may be asymptomatic at an early stage, and clinical symptoms may not present until hydatid cysts progress [4]. If inappropriately treated or untreated, a poor prognosis may be observed, with a post-surgical mortality rate of 2.2% and approximately 6.5% postoperative recurrence [5]. Cystic echinococcosis is widely prevalent across the world, and the incidence of human cystic echinococcosis is estimated to be more than 50/10^5^ in endemic foci, while the prevalence may be as high as 5% to 10% in Argentina, Peru, eastern Africa, Central Asia and China [6]. The global burden was estimated to be 184,000 disability adjusted life years (DALYs) due to cystic echinococcosis each year, resulting in a loss of USD 760 million a year [7]. In addition to huge economic burdens, this zoonotic parasitic disease also creates extremely high global public health burdens [8]. 

Currently, vaccination in livestock and deworming of dogs are the major interventions used for the prevention of cystic echinococcosis [9]. Since there are no remarkable cystic echinococcosis-induced pathological alterations in livestock, multisectoral collaboration is required for the implementation of integrated cystic echinococcosis control programs, which involves overcoming difficulties [10]. Although great strides have been achieved, multiple challenges remain to be faced before we can achieve the ambitious goal for cystic echinococcosis set in the WHO roadmap for neglected tropical diseases 2021–2030 [11]. Estimates of the disease burden due to cystic echinococcosis facilitate the progress towards eliminations; however, there has been little knowledge of the global morbidity, mortality and DALYs of cystic echinococcosis until now. This study aimed to collect the most up-to-date data about the global, regional and national disease burdens due to cystic echinococcosis from 1990 to 2019 and to project trends in the next 10 years.

## 2. Methods

### 2.1. Data Source

The incidence, age-standardized incidence rate (ASIR), mortality, age-standardized mortality rate (ASMR), DALYs and age-standardized DALY rate of cystic echinococcosis were captured in 204 countries from 1990 to 2019 based on the Global Burden of Disease Study 2019 (GBD 2019) data, which were retrieved from the Global Health Data Exchange tool (http://ghdx.healthdata.org/gbd-results-tool, assessed on 1 March 2023). The 204 countries were classified into 21 GBD regions and five socio-demographic index (SDI) quintiles (low, low-middle, middle, high-middle and high) based on SDI [12]. 

### 2.2. Statistical Analysis

The global, regional and national pooled number, deaths, DALYs, ASIR, ASMR and age-standardized DALY rate of cystic echinococcosis were estimated per 10^5^ population. The overall trends in the disease burden of cystic echinococcosis were evaluated using the estimated annual percent change (EAPC) from 1990 to 2019, and the trends in the regional and national ASIR of cystic echinococcosis from 1990 to 2019 were illustrated with a heat map. To identify factors affecting EAPC, we examined the associations of the EAPC and ASR of cystic echinococcosis with SDI, and a spline model was created to evaluate the association between the age-standardized DALY rate of cystic echinococcosis and SDI [13]. In addition, the disease burden due to cystic echinococcosis was projected using a Bayesian age-period-cohort analysis with integrated nested Laplace approximations from 2020 to 2030 [14]. All statistical analyses were performed using the R software version 4.1.2, and a *p* value of <0.05 was considered statistically significant.

## 3. Results

### 3.1. Global Trends in the Disease Burden of Cystic Echinococcosis

The global ASIR, ASMR and age-standardized DALY rate of cystic echinococcosis were 2.6/10^5^ [95% uncertainty interval (*UI*): (1.72/10^5^ to 3.7/10^5^)], 0.02/10^5^ [95% *UI*: (0.01/10^5^ to 0.02/10^5^)] and 1.56/10^5^ [95% *UI*: (1.14/10^5^ to 2.15/10^5^)] in 2019, and the global incident cases, deaths and DALYs of cystic echinococcosis were estimated to be 207,368 [95% *UI*: (6,347,183 to 8,769,520)], 1349 [95% *UI*: (987 to 1762)] and 122,457 [95% *UI*: (89,244 to 168,556)] in 2019, respectively (Table 1).

The global number of cystic echinococcosis cases increased from 134,980 [95% *UI*: (93,141 to 195,144)] in 1990 to 207,368 [95% *UI*: (137,807 to 303,233)] in 2019, with an EAPC of 0.54% [95% *UI*: (0.42 to 0.7)], while the global deaths from cystic echinococcosis reduced from 2839 [95% *UI*: (2218 to 3497)] in 1990 to 1349 [95% *UI*: (987 to 1762)] in 2019, with an EAPC of −0.52% [95% *UI*: (−0.66% to −0.34%)]. In addition, the global DALYs of cystic echinococcosis reduced from 210,044 [95% *UI*: (166,434 to 261,084)] in 1990 to 122,457 [95% *UI*: (89,244 to 168,556)] in 2019, with an EAPC of −0.42% [95% *UI*: (−0.57% to −0.23%)] (Table 2).

Although the global number of cystic echinococcosis cases appeared to rise from 1990 to 2019, the global ASIR of cystic echinococcosis appeared to decline [EAPC = −0.18%, 95% *UI*: (−0.24% to −0.12%)], and both the global ASMR [EAPC = −4.64%, 95% *UI*: (−4.85% to −4.43%)] and age-standardized DALY rate [EAPC = −3.38%, 95% *UI*: (−3.54% to −3.26%)] of cystic echinococcosis appeared to decline (Table 2).

### 3.2. Country-Specific ASIRs of Cystic Echinococcosis

**Figure 1:**
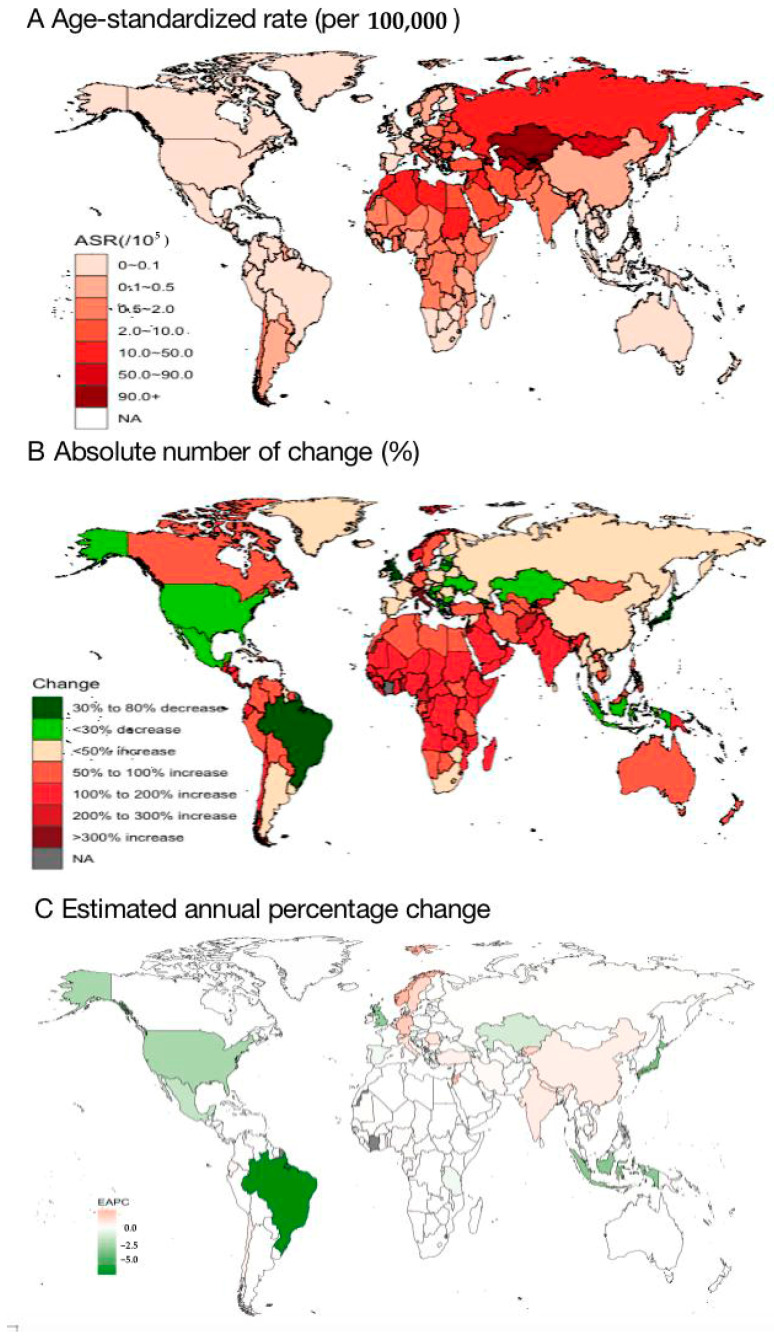
Global and national age-standardized incidences of cystic echinococcosis in 2019.

Across the 204 countries, the three highest ASIRs of cystic echinococcosis were measured in Kazakhstan [127.6/10^5^, 95% *UI*: (105.3/10^5^ to 153.8/10^5^)], Uzbekistan [123.5/10^5^, 95% *UI*: (58.7/10^5^ to 219.2/10^5^)] and Tajikistan [121.9/10^5^, 95% *UI*: (58.6/10^5^ to 213.9/10^5^)] from 1990 to 2019 (Figure 1A, Table S1). The largest increases in the number of incident cystic echinococcosis cases were seen in Italy (668.6% increase), Qatar (590.3% increase) and the United Arab Emirates (459.3% increase), and the largest reductions were seen in Brazil (79.2% reduction), the United Kingdom (64.5% reduction) and Japan (58.9% reduction) (Figure 1B, Table S1). In addition, the largest increases in the ASIRs of cystic echinococcosis were measured was observed in Norway [EAPC = 2.42%, 95% *UI*: (1.75% to 3.11%)], Jordan [EAPC = 2.06%, 95% *UI*: (1.66% to 2.47%)] and Germany [EAPC = 1.87%, 95% *UI*: (1.28% to 2.28%)], and the greatest reductions were seen in Brazil [EAPC = −6.65%, 95% UI: (−7.06% to −6.23%)], Japan [EAPC = −3.79%, 95% *UI*: (−4.57% to −3.01%)] and Indonesia [EAPC = −3.23%, 95% UI: (−3.82% to −2.64%)] (Figure 1C, Table S1).

### 3.3. Age-Specific Burdens of Cystic Echinococcosis

**Figure 2:**
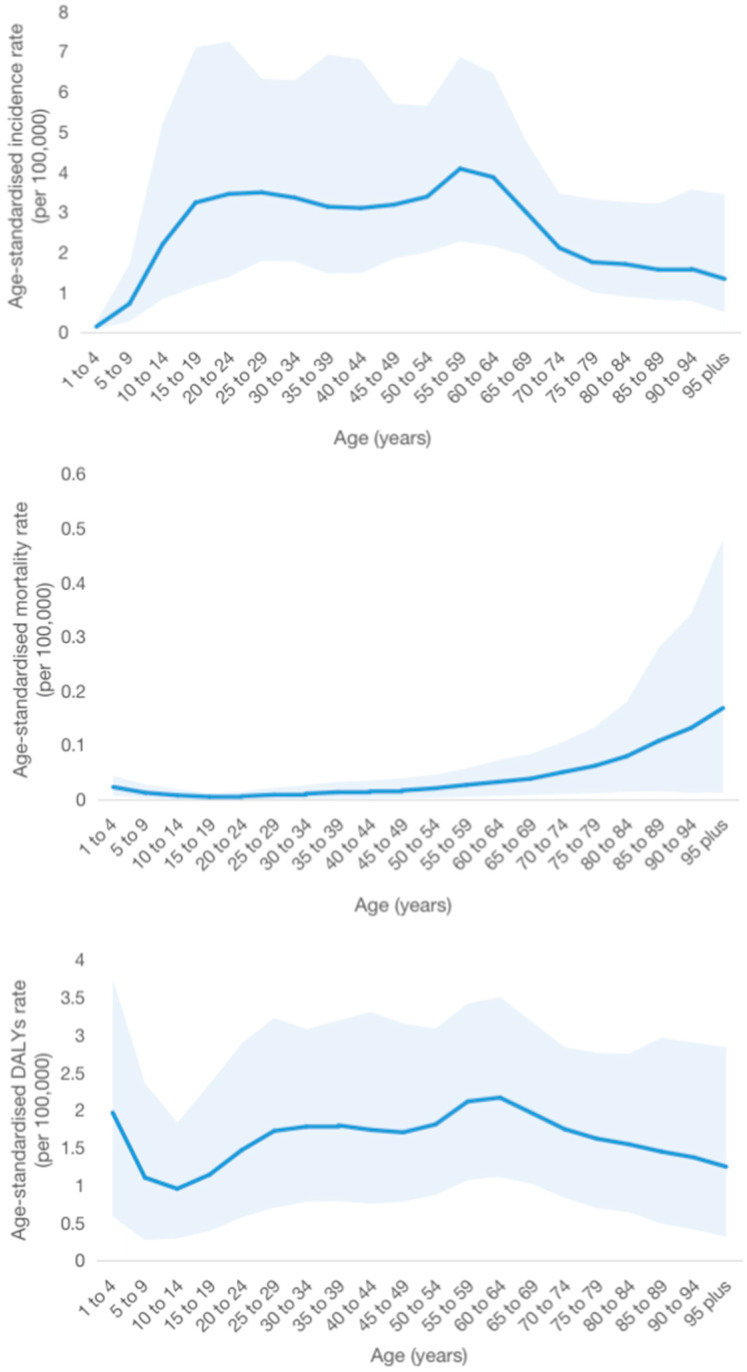
Global age-standardized incidence, mortality and DALYs of cystic echinococcosis in 2019.

The highest ASIR of cystic echinococcosis was found at the ages of 50 to 59 years in 2019 [4.08/10^5^, 95% *UI*: (2.27/10^5^ to 6.86/10^5^)], followed by the ages of 25 to 29 years [3.49/10^5^, 95% *UI*: (1.79/10^5^ to 6.32/10^5^)], and the ASMR of cystic echinococcosis increased with age, with the highest seen at ages of 95 years and older [0.17/10^5^, 95% *UI*: (0.01/10^5^ to 0.48/10^5^)]. In addition, the greatest age-standardized DALY rate of cystic echinococcosis was measured at the ages of 60 to 64 years [2.17/10^5^, 95% *UI*: (1.11/10^5^ to 3.51/10^5^)], followed by the ages of 1 to 4 years [1.97/10^5^, 95% *UI*: (0.59/10^5^ to 3.75/10^5^)] (Figure 2).

### 3.4. Association between Age-Specific Burdens of Cystic Echinococcosis and SDI

**Figure 3:**
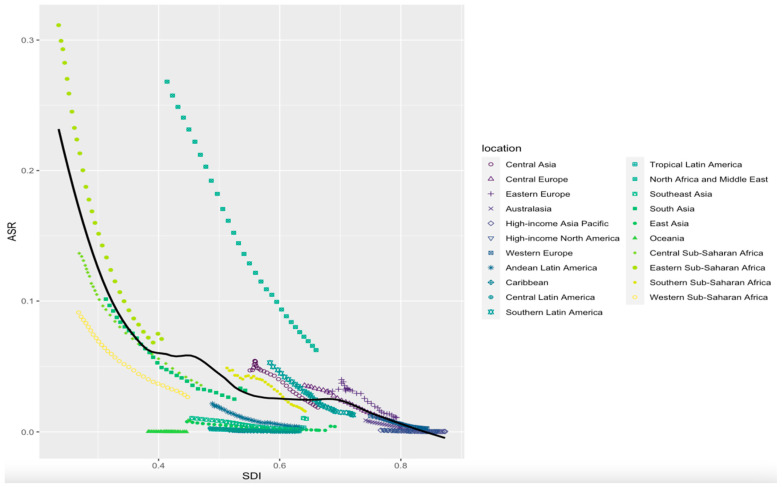
Association between age-standardized mortality for cystic echinococcosis and SDI in 21 GBD regions from 1990 to 2019.

**Figure 4:**
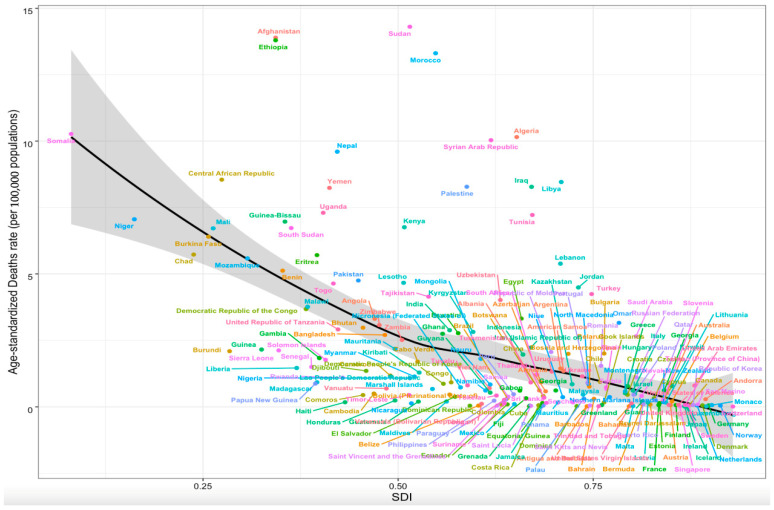
Association between age-standardized mortality of cystic echinococcosis and SDI in 204 countries in 2019.

The ASMR of cystic echinococcosis was found to correlate negatively with the SDI in 21 GBD regions from 1990 to 2019 (*R* = −0.544, *p* < 0.01). The ASMRs of cystic echinococcosis in high-SDI regions and Australia were similar to those expected, while the ASMRs of cystic echinococcosis in moderate-SDI regions differed greatly from those expected (Figure 3). The ASMR of cystic echinococcosis was found to correlate negatively with the SDI in 204 countries in 2019 (*R* = −0.546, *p* < 0.01), and the ASMRs of cystic echinococcosis were much higher than those expected in a few middle-SDI countries (Figure 4). In addition, there were no associations found between the ASIR and age-standardized DALY rate of cystic echinococcosis and the SDI (Figures S1 and S2).

### 3.5. Projections of the Global Burden Due to Cystic Echinococcosis from 2020 to 2030

**Figure 5:**
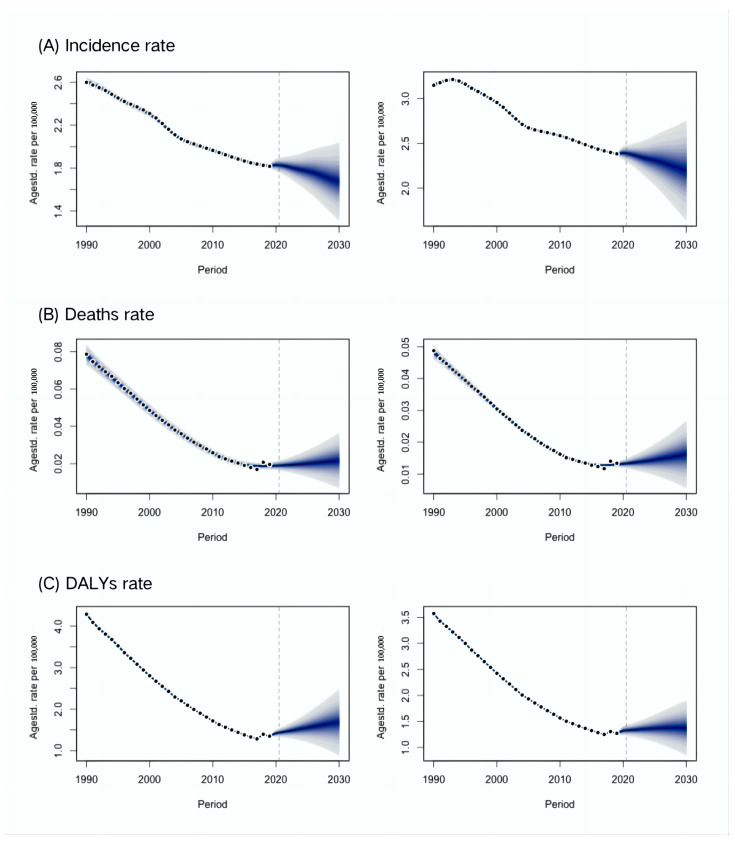
Trends in global age-standardized incidence, mortality and DALYs of cystic echinococcosis from 1990 to 2019 and projections from 2020 to 2030. Left, men; right, women.

The global ASIR of cystic echinococcosis is projected to decline in both men and women from 2020 to 2030 based on a Bayesian age-period-cohort analysis with integrated nested Laplace approximations from 2020 to 2030; however, a slight rise is projected in the global ASMR and age-standardized DALY rate of cystic echinococcosis (Figure 5).

## 4. Discussion

In the current study, we assessed the global disease burden due to cystic echinococcosis and measured the incidence, mortality and DALY rate of cystic echinococcosis in 204 countries during the period from 1990 to 2019. A total of 207,368 cystic echinococcosis cases were reported across 204 countries in 2019, which was significantly higher than in 1990 (134,980 cases). Our data showed that the global incidence, mortality and DALY rate of cystic echinococcosis all appeared to decline during the 30-year period from 1990 to 2019. However, the ASIR of cystic echinococcosis appeared to rise in low-, low-middle-, middle- and high-SDI regions, while the ASMR and age-standardized DALY rate of cystic echinococcosis remained in decline in all five SDI regions. This demonstrates that understanding the trends in the epidemiology and disease burden of cystic echinococcosis is of critical significance for public health officials and policy makers if they are to appropriately allocate medical resources.

In this study, we charted a continuous reduction in the global ASIR of cystic echinococcosis from 1990 to 2019, which we mainly attribute to the reduction in the global ASIR of cystic echinococcosis in the middle-high-SDI region. In addition, we found that the long-standing implementation of national echinococcosis control programs has resulted in a remarkable reduction in the burden of cystic echinococcosis in some countries in South America (Uruguay, Chile and Argentina) and China [15,16,17]. In this study, the largest increase in the global ASIR of cystic echinococcosis was measured in the high-SDI region, and Norway, Jordan and Germany were the three countries with the largest increases. A systematic review of the scientific and gray literature showed a remarkable rise in the incidence of cystic echinococcosis in Norway and Germany from 1997 to 2021 [18]. The increase in the incidence of cystic echinococcosis in low-endemic regions may be partly attributed to the numbers of immigrants from Syria and Afghanistan, where cystic echinococcosis is highly prevalent [18]; international travel; and physicians’ increased knowledge about cystic echinococcosis control [14]. Therefore, the epidemics of cystic echinococcosis in low-prevalence regions cannot be neglected [19], where we recommend launching national echinococcosis control programs [20].

In this study, we found a large reduction in the global ASMR and age-standardized DALY rate of cystic echinococcosis from 1990 to 2019 relative to the ASIR. This may be attributed to the release of the roadmaps for neglected tropical diseases by the WHO in 2012 and 2020 [21,22], and the signing of the London Declaration on Neglected Tropical Diseases in 2012 [23], which increased the emphasis on neglected tropical diseases worldwide, including echinococcosis. Furthermore, in 2009, an expert consensus on the diagnosis and treatment of cystic and alveolar echinococcosis in humans was reached by the WHO-Informal Working Group on Echinococcosis (WHO-IWGE) [24]. This consensus outlined the diagnostic and treatment options for echinococcosis, which facilitated a reduction in the mortality and disease burden of echinococcosis. In addition, the launch of active surveillance of cystic echinococcosis through ultrasound and treatment of asymptomatic patients with albendazole also contributed to reductions in mortality from cystic echinococcosis in endemic foci (for example, Rio Negro, Argentina) [25]. Moreover, recently, the WHO has collaborated with veterinary and food security administrations to support the formulation of echinococcosis control programs under the One Health framework, which is of great importance for reducing the disease burden of echinococcosis and protecting the food value chain [26,27,28].

Based on GBD2019 data, we found a higher ASIR and age-standardized DALY rate of cystic echinococcosis among women than among men, which was consistent with previous reports [13]. A recent meta-analysis showed that being female is a potential risk factor of human cystic echinococcosis [29]. This may be because women mainly work on feeding dogs, grazing animals, milking and collecting stools in many regions, resulting in a high risk of contamination with *Echinococcus* eggs [30,31,32]. Therefore, a higher priority for the management of cystic echinococcosis should be given to women. In addition, a high incidence rate of cystic echinococcosis was seen in individuals at the ages of 55 to 65 years. This may be because people at the ages of 55 to 65 years are most active in animal husbandry [33], or because cystic echinococcosis is a chronic infectious disease, which has a long course of disease. The clinical symptoms may present 5 to 20 years following *Echinococcus* infection, meaning individuals at the ages of 55 to 65 years may have been exposed to settings contaminated by *Echinococcus* eggs released from infected hosts for long periods of time, and they may be showing symptoms that have arisen following a long incubation period [30].

In this study, we found that the ASMR of cystic echinococcosis showed a trend of reduction with the SDI in the 21 GBD regions and 204 countries, and there were no significant associations of the SDI with the ASIR or age-standardized DALY rate of cystic echinococcosis. In regions with a higher SDI, better hygiene conditions and higher levels of urbanization lead to a lower risk of exposure to echinococcosis. Education is an indicator of SDI, and a lower educational level is observed in lower-SDI regions. Furthermore, it has been reported that echinococcosis is highly prevalent in underdeveloped communities with low education levels and literacy rates or resource-poor communities [34]. Therefore, health education pertaining to knowledge of echinococcosis control is of great importance for disease prevention and control. Nevertheless, easily readable health education materials are required given the low education level, in order to improve the acceptability and compliance [35]. In addition to health education, the management of dogs, vaccination of sheep, access to safe water and case detection and treatment are recommended for cystic echinococcosis control [1,2,5].

In this study, we evaluated the temporal trends in the global disease burden of cystic echinococcosis from 1990 to 2019 and projected trends in the next 10 years based on the GBD2019 data. Our data showed a continuous decline in the ASIR, ASMR and age-standardized DALY rate of cystic echinococcosis during the 30-year period from 1990 to 2019, and the global ASIR of cystic echinococcosis is projected to decline from 2020 to 2030, which may be attributed to the global strengthening of echinococcosis prevention and control following the release of the WHO roadmap for neglected tropical diseases 2021–2030. In addition, the global ASMR and age-standardized DALY rate of cystic echinococcosis are projected to rise slightly, which may be attributed to the alteration of the population structure and lack of timely disease identification and treatment. Population ageing is one of the most significant trends of the 21st century, and the number of people aged 60 years and over is projected to rise from 1 billion in 2020 to 1.4 billion in 2030 [36]. Meanwhile, cystic echinococcosis has a long course of disease and a low rate of early diagnosis, meaning most cases are diagnosed at an advanced age [37,38,39,40]. 

This study had some limitations. First, the source, analysis and assessment of the GBD2019 data were adjusted many times to improve the data accuracy and comparability; however, there was inevitable bias in the integrity and accuracy of the GBD2019 data. Second, there are no available disability weights of cystic echinococcosis in the GBD2019 data, and so the disability weights of liver cancer were employed in this study, which may have introduced bias to the study results. Further studies that overcome these limitations are required to validate the findings from this study.

In summary, our findings demonstrate the global disease burden of cystic echinococcosis from 1990 to 2019 and project the future trends in the next 10 years. Our data offer evidence that public health officials and policy makers may use to formulate and implement cost–benefit interventions to tackle the disease burden attributable to cystic echinococcosis. We recommend that more health resources should be allocated to low-SDI regions, women and the elderly aged 55 to 65 years to reduce the disease burden caused by cystic echinococcosis, and sustainable, integrated interventions based on the One Health concept are needed if we are to achieve the elimination of cystic echinococcosis from this wormy world [41,42].

## Figures and Tables

**Table 1:** Comparison of global incidence and age-standardized incidence, mortality and age-standardized mortality and DALYs and age-standardized DALYs of cystic echinococcosis between 1990 and 2019.

Items	Incidence				Mortality				DALYs			
	Number (95% CI)		ASR, per 10^5^ (95% CI)		Number (95% CI)		ASR, per 10^5^ (95% CI)		Number (95% CI)		ASR, per 10^5^ (95% CI)	
	1990	2019	1990	2019	1990	2019	1990	2019	1990	2019	1990	2019
Global	134,980 (93,141–195,144)	207,368 (137,807–303,233)	2.65 (1.87–3.7)	2.6 (1.72–3.79)	2839 (2218–3497)	1349 (987–1762)	0.06 (0.04–0.07)	0.02 (0.01–0.02)	210,044 (166,434–261,084)	122,457 (89,244–168,556)	3.82 (3.05–4.7)	1.56 (1.14–2.15)
Gender												
Male	55,004 (36,334–84,605)	83,318 (52,851–127,144)	2.12 (1.43–3.1)	2.09 (1.34–3.21)	1480 (1105–1879)	726 (455–1030)	0.06 (0.04–0.08)	0.02 (0.01–0.03)	103,864 (78,388–133,209)	58,532 (40,465–81,788)	3.74 (2.86–4.75)	1.49 (1.04–2.1)
Female	79,977 (56,335–113,027)	124,050 (84,455–175,220)	3.17 (2.28–4.31)	3.1 (2.1–4.38)	1359 (995–1776)	623 (385–879)	0.05 (0.04–0.07)	0.02 (0.01–0.02)	103,864 (78,388–133,209)	63,925 (43,062–87,764)	3.9 (2.99–4.94)	1.62 (1.08–2.25)
Socio-demographic index												
Low	5079 (3391–7571)	12,430 (8169–18,540)	1.11 (0.78–1.61)	1.2 (0.83–1.71)	1019 (739–1378)	438 (303–592)	0.2 (0.15–0.26)	0.05 (0.04–0.07)	70,767 (50,134–97,271)	27,923 (18,880–38,026)	10.91 (8.1–14.56)	2.58 (1.88–3.36)
Low-middle	21,426 (12,586–34,881)	41,908 (25,494–64,714)	2.17 (1.36–3.3)	2.37 (1.47–3.62)	1000 (798–1230)	455 (338–587)	0.1 (0.08–0.13)	0.03 (0.02–0.04)	67,621 (53,616–85,566)	31,737 (23,685–42,274)	5.62 (4.58–6.86)	1.87 (1.41–2.43)
Middle	48,647 (24,709–87,978)	85,224 (45,368–144,835)	2.95 (1.58–4.89)	3.38 (1.8–5.84)	437 (333–537)	276 (195–373)	0.03 (0.02–0.04)	0.01 (0.01–0.02)	38,780 (27,433–54,650)	36,915 (22,015–63,062)	2.35 (1.73–3.23)	1.45 (0.86–2.47)
High-middle	58,010 (47,824–69,658)	64,390 (53,694–76,666)	4.96 (4.1–5.92)	4.03 (3.35–4.8)	348 (244–463)	161 (112–220)	0.03 (0.02–0.04)	0.01 (0.01–0.01)	30,961 (23,984–39,200)	24,106 (17,621–32,569)	2.7 (2.1–3.39)	1.46 (1.05–1.98)
High	1799 (1083–2828)	3359 (1985–5352)	0.21 (0.13–0.35)	0.34 (0.19–0.54)	34 (20–51)	19 (11–28)	0 (0–0.01)	0 (0–0)	1849 (1287–2529)	1733 (1123–2638)	0.22 (0.15–0.3)	0.16 (0.1–0.25)
GBD region												
High-income Asia Pacific	93 (35–254)	51 (33–91)	0.05 (0.02–0.14)	0.03 (0.01–0.05)	2 (1–4)	1 (1–2)	0 (0–0)	0 (0–0)	127 (79–201)	42 (27–61)	0.07 (0.04–0.11)	0.02 (0.01–0.03)
Central Asia	65,909 (43,716–102,773)	95102 (56,969–152,588)	103.28 (68.82–152.04)	100.32 (61.06–159.16)	27 (17–38)	15 (9–21)	0.05 (0.03–0.07)	0.02 (0.01–0.03)	21,147 (12,039–36,064)	30,110 (15,271–54,362)	33.74 (19.48–56.19)	31.68 (16.44–56)
East Asia	5072 (1560–11,800)	6793 (3000–13,654)	0.4 (0.13–0.88)	0.45 (0.18–0.94)	78 (65–92)	74 (61–88)	0.01 (0.01–0.01)	0 (0–0)	5541 (4037–8534)	4395 (2897–7336)	0.47 (0.35–0.67)	0.26 (0.17–0.45)
South Asia	7321 (4559–13,128)	14,984 (9539–28,274)	0.8 (0.51–1.52)	0.86 (0.56–1.64)	930 (727–1162)	478 (340–630)	0.1 (0.08–0.13)	0.03 (0.02–0.04)	59,185 (46,325–73,797)	23,798 (17,893–30,900)	4.99 (3.96–6.16)	1.39 (1.05–1.8)
Southeast Asia	664 (164–2059)	574 (163–1533)	0.14 (0.04–0.38)	0.08 (0.02–0.23)	39 (25–54)	61 (37–89)	0.01 (0.01–0.01)	0.01 (0.01–0.01)	2265 (1534–3264)	2460 (1557–3556)	0.5 (0.34–0.7)	0.37 (0.24–0.52)
Australasia	5 (4–7)	9 (7–13)	0.02 (0.02–0.03)	0.02 (0.02–0.03)	2 (1–3)	1 (0–1)	0.01 (0–0.01)	0 (0–0)	60 (35–88)	23 (15–33)	0.28 (0.16–0.4)	0.06 (0.04–0.09)
Caribbean	18 (14–24)	22 (17–28)	0.06 (0.04–0.07)	0.04 (0.04–0.06)	0 (0–1)	0 (0–1)	0 (0–0)	0 (0–0)	33 (16–56)	22 (15–31)	0.09 (0.05–0.15)	0.05 (0.03–0.07)
Central Europe	2279 (1879–2734)	2326 (1988–2686)	1.74 (1.41–2.1)	1.76 (1.49–2.06)	48 (30–69)	15 (8–24)	0.04 (0.02–0.05)	0.01 (0–0.01)	2313 (1716–2976)	1166 (847–1534)	1.74 (1.29–2.23)	0.81 (0.59–1.08)
Eastern Europe	23,796 (19,659–28,303)	24,583 (20,301–29,406)	9.48 (7.9–11.19)	9.68 (8.14–11.47)	79 (43–119)	32 (17–50)	0.03 (0.02–0.05)	0.01 (0.01–0.02)	10,065 (7392–13,113)	8648 (6168–11,751)	3.99 (2.94–5.19)	3.31 (2.36–4.51)
Western Europe	870 (657–1242)	4635 (3933–5435)	0.21 (0.16–0.31)	0.86 (0.73–1.02)	63 (37–90)	24 (13–38)	0.01 (0.01–0.02)	0 (0–0)	1997 (1343–2741)	1764 (1303–2337)	0.45 (0.3–0.6)	0.31 (0.22–0.4)
Andean Latin America	7 (5–10)	13 (10–16)	0.02 (0.02–0.03)	0.02 (0.02–0.03)	7 (4–10)	2 (1–3)	0.02 (0.01–0.03)	0 (0–0.01)	394 (193–630)	85 (48–133)	0.99 (0.55–1.49)	0.14 (0.08–0.21)
Central Latin America	92 (34–256)	91 (50–188)	0.06 (0.02–0.17)	0.04 (0.02–0.08)	3 (2–5)	3 (2–4)	0 (0–0)	0 (0–0)	220 (114–341)	133 (83–194)	0.14 (0.08–0.21)	0.05 (0.03–0.08)
Southern Latin America	336 (279–400)	569 (483–666)	0.69 (0.58–0.82)	0.79 (0.67–0.93)	24 (16–33)	10 (6–15)	0.05 (0.04–0.07)	0.01 (0.01–0.02)	949 (669–1236)	476 (345–619)	1.97 (1.39–2.57)	0.65 (0.47–0.84)
Tropical Latin America	816 (265–2027)	192 (112–346)	0.63 (0.23–1.74)	0.08 (0.05–0.15)	2 (1–3)	8 (4–12)	0 (0–0)	0 (0–0)	349 (170–748)	332 (206–469)	0.27 (0.14–0.64)	0.14 (0.09–0.2)
North Africa and Middle East	23,763 (14,395–37,029)	48,205 (29,717–72,394)	7.61 (4.76–11.23)	7.81 (4.98–11.44)	689 (541–854)	287 (211–374)	0.27 (0.21–0.33)	0.06 (0.05–0.08)	45,756 (35,616–58,030)	26,631 (18,912–37,908)	13.58 (10.88–16.64)	4.53 (3.3–6.27)
High-income North America	130 (74–239)	118 (75–193)	0.04 (0.02–0.08)	0.03 (0.02–0.04)	5 (3–8)	4 (2–6)	0 (0–0)	0 (0–0)	202 (129–289)	154 (99–214)	0.07 (0.04–0.1)	0.04 (0.02–0.05)
Oceania	0 (0–1)	1 (0–1)	0 (0–0.01)	0 (0–0.01)	0 (0–0)	1 (0–1)	0 (0–0)	0.01 (0–0.01)	0 (0–1)	42 (22–68)	0.01 (0.01–0.01)	0.34 (0.19–0.53)
Central Sub-Saharan Africa	811 (518–1201)	2030 (1294–3011)	1.69 (1.18–2.33)	1.7 (1.18–2.34)	71 (50–99)	29 (19–41)	0.14 (0.09–0.18)	0.04 (0.02–0.05)	5105 (3621–7210)	2047 (1430–2771)	7.45 (5.35–10.23)	1.76 (1.28–2.33)
Eastern Sub-Saharan Africa	1477 (1124–1890)	3260 (2485–4157)	0.81 (0.64–1.01)	0.8 (0.63–0.99)	558 (383–784)	191 (130–266)	0.31 (0.22–0.42)	0.07 (0.05–0.1)	38,989 (26,530–56,175)	11,813 (7615–16,493)	16.61 (11.77–23.09)	3.04 (2.1–4.1)
Southern Sub-Saharan Africa	20 (15–27)	29 (22–38)	0.04 (0.03–0.05)	0.04 (0.03–0.05)	20 (11–29)	10 (6–15)	0.05 (0.03–0.07)	0.02 (0.01–0.02)	1074 (540–1686)	456 (246–695)	2.15 (1.2–3.21)	0.6 (0.34–0.9)
Western Sub-Saharan Africa	1499 (941–2276)	3781 (2342–5797)	0.88 (0.59–1.25)	0.91 (0.61–1.29)	191 (117–283)	103 (60–157)	0.09 (0.06–0.13)	0.03 (0.02–0.04)	14,274 (8226–22,125)	7858 (4459–12,066)	5.46 (3.48–7.96)	1.54 (0.99–2.2)

**Table 2:** Comparison of global incidence, mortality and DALYs of cystic echinococcosis between 1990 and 2019.

Items	Incidence		Mortality		DALYs	
	Annual Rate of Change (%, 95% CI)	EAPC (%, 95% CI)	Annual Rate of Change (%, 95% CI)	EAPC (%, 95% CI)	Annual Rate of Change (%, 95% CI)	EAPC (%, 95% CI)
Global	0.54 (0.42–0.7)	−0.18 (−0.24–0.12)	−0.52 (−0.66–0.34)	−4.64 (−4.85–4.43)	−0.42 (−0.57–0.23)	−3.38 (−3.5–3.26)
Gender						
Male	0.51 (0.39–0.7)	−0.08 (−0.13–0.02)	−0.51 (−0.7–0.24)	−4.54 (−4.75–4.32)	−0.44 (−0.62–0.21)	−3.47 (−3.6–3.35)
Female	0.55 (0.43–0.71)	−0.24 (−0.31–0.18)	−0.54 (−0.73–0.27)	−4.78 (−4.99–4.57)	−0.4 (−0.58–0.15)	−3.3 (−3.43–3.18)
Socio-demographic index						
Low	1.39 (1.26–1.51)	0.24 (0.22–0.26)	−0.57 (−0.71–0.37)	−4.81 (−4.97–4.65)	−0.61 (−0.74–0.41)	−5.18 (−5.31–5.06)
Low-middle	0.94 (0.76–1.16)	0.38 (0.35–0.42)	−0.54 (−0.67–0.38)	−4.81 (−5.08–4.54)	−0.53 (−0.66–0.37)	−4.16 (−4.37–3.96)
Middle	0.74 (0.49–1.03)	0.48 (0.44–0.51)	−0.37 (−0.58–0.08)	−4.64 (−5.08–4.2)	−0.05 (−0.32–0.24)	−1.94 (−2.14–1.74)
High-middle	0.11 (0.05–0.17)	−1.04 (−1.19–0.89)	−0.54 (−0.71–0.3)	−5.17 (−5.37–4.96)	−0.22 (−0.35–0.08)	−2.51 (−2.66–2.35)
High	0.87 (0.52–1.36)	1.67 (1.54–1.81)	−0.45 (−0.71–0.05)	−4.01 (−4.17–3.86)	−0.06 (−0.39–0.38)	−1.14 (−1.34–0.94)
GBD region						
High-income Asia Pacific	−0.45 (−0.66–0.02)	−2.9 (−3.51–2.28)	−0.52 (−0.78–0.02)	−6.76 (−7.52–6)	−0.67 (−0.79–0.46)	−5.66 (−6.32–5.01)
Central Asia	0.44 (0.28–0.67)	−0.2 (−0.28–0.12)	−0.45 (−0.7–0.01)	−3.61 (−4.11–3.11)	0.42 (0.22–0.68)	−0.35 (−0.42–0.27)
East Asia	0.34 (−0.05–1.06)	0.56 (0.49–0.64)	−0.05 (−0.27–0.24)	−5.35 (−6.64–4.05)	−0.21 (−0.38–0.04)	−2.77 (−3.28–2.26)
South Asia	1.05 (0.87–1.25)	0.32 (0.25–0.39)	−0.49 (−0.64–0.3)	−4.74 (−5.05–4.42)	−0.6 (−0.71–0.46)	−4.92 (−5.18–4.67)
Southeast Asia	−0.14 (−0.32–0.21)	−2.35 (−2.77–1.94)	0.59 (−0.09–1.76)	−4.35 (−5.96–2.72)	0.09 (−0.37–0.93)	−4.66 (−6.03–3.26)
Australasia	0.76 (0.61–0.89)	0.01 (−0.06–0.07)	−0.56 (−0.79–0.1)	−5.42 (−5.56–5.29)	−0.62 (−0.79–0.27)	−5.26 (−5.42–5.1)
Caribbean	0.18 (0.06–0.32)	−0.73 (−0.78–0.68)	−0.17 (−0.6–0.82)	−2.98 (−3.59–2.37)	−0.33 (−0.65–0.41)	−2.69 (−3.04–2.35)
Central Europe	0.02 (−0.05–0.1)	0.09 (−0.02–0.19)	−0.69 (−0.85–0.43)	−5.52 (−5.72–5.33)	−0.5 (−0.64–0.29)	−2.86 (−2.95–2.77)
Eastern Europe	0.03 (−0.02–0.08)	0.09 (0.08–0.09)	−0.6 (−0.81–0.2)	−4.39 (−5.01–3.76)	−0.14 (−0.27–0.01)	−0.92 (−1.07–0.77)
Western Europe	4.33 (3.04–5.61)	0.45 (−0.87–1.79)	−0.62 (−0.81–0.25)	−5.2% (−5.31–5.15)	−0.12 (−0.42–0.43)	−2.49 (−2.85–2.13)
Andean Latin America	0.78 (0.6–0.99)	−0.12 (−0.13–0.12)	−0.68 (−0.84–0.37)	−5.98 (−6.1–5.85)	−0.78 (−0.9–0.51)	−6.71 (−6.82–6.6)
Central Latin America	−0.02 (−0.33–0.62)	−1.28 (−1.8–0.75)	−0.21 (−0.6–0.54)	−4.16 (−4.67–3.65)	−0.39 (−0.66–0.2)	−3.97% (−4.43–3.51)
Southern Latin America	0.69 (0.61–0.78)	0.3 (−0.02–0.63)	−0.58 (−0.76–0.31)	−4.77 (−4.84–4.7)	−0.5 (−0.66–0.23)	−3.75% (−3.79–3.7)
Tropical Latin America	−0.77 (−0.85–0.56)	−6.29 (−6.7–5.87)	2.65 (0.82–6.16)	−2.7 (−4.52–0.84)	−0.05 (−0.55–1.14)	−4.5% (−5.7–3.29)
North Africa and Middle East	1.03 (0.81–1.29)	0.03 (−0.01–0.08)	−0.58 (−0.7–0.43)	−5.09 (−5.17–5.01)	−0.42 (−0.59–0.2)	−3.91% (−4–3.83)
High-income North America	−0.09 (−0.3–0.17)	−1.95 (−2.38–1.53)	−0.19 (−0.6–0.64)	−3.54 (−4.06–3.01)	−0.24 (−0.55–0.29)	−3.07% (−3.55–2.59)
Oceania	0.9 (0.58–1.2)	−0.19 (−0.22–0.16)	108.72 (52.51–219.73)	0.62 (−4.47–5.98)	92.41 (41.29–182.94)	2.01% (−2.69–6.94)
Central Sub-Saharan Africa	1.5 (1.42–1.59)	0.04 (0.03–0.04)	−0.59 (−0.74–0.37)	−4.65 (−4.95–4.36)	−0.6 (−0.73–0.4)	−5.02% (−5.3–4.74)
Eastern Sub-Saharan Africa	1.21 (1.16–1.26)	−0.07 (−0.1–0.03)	−0.66 (−0.78–0.48)	−5.54 (−5.73–5.34)	−0.7 (−0.81–0.52)	−6.19% (−6.36–6.03)
Southern Sub-Saharan Africa	0.45 (0.36–0.58)	−0.05 (−0.07–0.03)	−0.48 (−0.74–0.02)	−3.8 (−4.24–3.35)	−0.58 (−0.8–0.07)	−4.27% (−4.72–3.82)
Western Sub-Saharan Africa	1.52 (1.46–1.58)	0.12 (0.1–0.14)	−0.46 (−0.71–0.03)	−4.2 (−4.29–4.11)	−0.45 (−0.72–0.02)	−4.31% (−4.39–4.23)

## Data Availability

All data presented in this study are available upon request by contact with the corresponding author.

## Supplementary Materials

The following supporting information can be downloaded at https://www.mdpi.com/article/10.3390/tropicalmed9040087/s1, Figure S1: Association between age–standardized incidence of cystic echinococcosis and SDI in 21 GBD regions from 1990 to 2019; Figure S2: Association between age–standardized DALY rate of cystic echinococcosis and SDI in 21 GBD regions from 1990 to 2019; Table S1: Incidence of Cystic echinococcosis in 1990 and 2019 in 204 countries, with EAPC from 1990 and 2019.
