# Supplementary material for: Global, Regional and National Burden of Human Cystic Echinococcosis from 1990 to 2019: A Systematic Analysis for the Global Burden of Disease Study 2019
Source: Trop Med Infect Dis. 2024 Apr 17;9(4):87. doi: 10.3390/tropicalmed9040087 (PMC11054543; doi:10.3390/tropicalmed9040087)
Supplement: Supplementary file 1 [file tropicalmed-09-00087-s001.zip › tropicalmed-2840992-supplementary.pdf]

Supplementary Materails

Table S1. Incidence of Cystic echinococcosis in 1990 and 2019 in 204 countries, with EAPC from 1990 and 2019.

location	Num_1990	ASR_1990	Num_2019	ASR_2019	Num_change	EAPC_CI
Afghanistan	527 (294–872)	5.29 (2.93–8.4)	1759 (878–3063)	5.24 (2.91–8.39)	2.34% (1.78–2.68)	−0.08% (−0.1–−0.06)
Albania	35 (26–45)	1.13 (0.88–1.4)	35 (28–43)	1.14 (0.88–1.4)	0% (−0.12–0.15)	0.01% (0.01–0.01)
Algeria	2987 (1546–5032)	13.09 (7.34–21.02)	5586 (3001–9111)	13.17 (7.32–20.72)	0.87% (0.48–1.36)	−0.02% (−0.04–0)
American Samoa	0 (0–0)	0.07 (0.05–0.09)	0 (0–0)	0.07 (0.05–0.09)	0.19% (0.1–0.29)	0.12% (0.1–0.14)
Andorra	0 (0–0)	0.42 (0.17–0.81)	0 (0–1)	0.41 (0.17–0.78)	0.65% (0.19–1.23)	0.02% (0.01–0.04)
Angola	158 (100–232)	1.77 (1.23–2.45)	471 (301–688)	1.79 (1.24–2.46)	1.98% (1.87–2.11)	0.06% (0.05–0.06)
Antigua and Barbuda	0 (0–0)	0.01 (0.01–0.02)	0 (0–0)	0.01 (0.01–0.02)	0.48% (0.26–0.72)	−0.06% (−0.02—0.09)
Argentina	177 (143–216)	0.54 (0.44–0.66)	256 (207–310)	0.54 (0.43–0.65)	0.45% (0.37–0.52)	0% (0–0.01)
Armenia	1902 (873–3500)	56.48 (26.55–101.85)	1849 (862–3383)	56.11 (25.8–100.3)	−0.03% (−0.21–0.21)	−0.01% (−0.03–0)
Australia	4 (3–5)	0.02 (0.02–0.03)	7 (5–10)	0.02 (0.02–0.03)	0.78% (0.68–0.87)	0% (0–0)
Austria	30 (25–35)	0.35 (0.29–0.42)	31 (26–37)	0.32 (0.26–0.39)	0.05% (−0.03–0.14)	−0.41% (−0.67—0.15)
Azerbaijan	3958 (1726–7618)	56.26 (25.42–99.52)	6198 (2739–11638)	55.83 (25.38–102.43)	0.57% (0.25–0.9)	−0.02% (−0.04—0.01)
Bahamas	0 (0–0)	0.01 (0.01–0.02)	0 (0–0)	0.01 (0.01–0.02)	0.53% (0.3–0.84)	−0.07% (−0.1—0.03)
Bahrain	3 (1–6)	0.65 (0.31–1.14)	11 (5–21)	0.66 (0.31–1.18)	2.21% (1.43–3.4)	0.02% (0–0.04)
Bangladesh	1466 (305–4270)	1.76 (0.42–5.59)	2735 (618–8823)	1.77 (0.43–5.74)	0.87% (0.38–1.54)	0.01% (−0.01–0.03)
Barbados	0 (0–0)	0.01 (0.01–0.02)	0 (0–0)	0.01 (0.01–0.02)	0.21% (0.03–0.44)	−0.08% (−0.11—0.06)
Belarus	763 (625–918)	6.57 (5.43–7.91)	765 (621–919)	6.53 (5.41–7.8)	0% (−0.07–0.08)	−0.01% (−0.01—0.01)
Belgium	5 (4–6)	0.04 (0.03–0.05)	8 (7–10)	0.06 (0.05–0.08)	0.74% (0.61–0.92)	1.12% (0.81–1.43)
Belize	0 (0–0)	0.01 (0.01–0.02)	0 (0–0)	0.01 (0.01–0.02)	1.26% (0.98–1.63)	−0.07% (−0.11—0.03)
Benin	62 (40–92)	1.54 (1.05–2.15)	175 (110–262)	1.55 (1.05–2.16)	1.82% (1.71–1.95)	0.06% (0.04–0.07)

location	Num_1990	ASR_1990	Num_2019	ASR_2019	Num_change	EAPC_CI
Bermuda	0 (0–0)	0.29 (0.23–0.37)	0 (0–0)	0.29 (0.23–0.36)	0.2% (0.05–0.37)	−0.06% (−0.08—0.03)
Bhutan	8 (2–24)	1.78 (0.44–5.55)	13 (3–39)	1.74 (0.41–5.46)	0.51% (0.09–1.05)	−0.16% (−0.19—0.12)
Bolivia (Plurinational State of)	0 (0–1)	0.01 (0–0.01)	1 (0–1)	0.01 (0–0.01)	0.96% (0.71–1.34)	−0.03% (−0.04—0.02)
Bosnia and Herzegovina	147 (116–182)	3.16 (2.5–3.87)	122 (97–152)	3.13 (2.47–3.89)	−0.17% (−0.25—0.07)	−0.03% (−0.03—0.02)
Botswana	0 (0–0)	0 (0–0.01)	0 (0–0)	0 (0–0.01)	0.7% (0.44–1.12)	0.01% (0.01–0.01)
Brazil	804 (255–2013)	0.64 (0.23–1.77)	167 (89–321)	0.07 (0.04–0.14)	−0.79% (−0.87—0.62)	−6.65% (−7.06—6.23)
Brunei Darussalam	0 (0–0)	0.01 (0–0.01)	0 (0–0)	0.01 (0–0.01)	0.76% (0.52–1.13)	0.01% (0.01–0.01)
Bulgaria	305 (243–374)	3.15 (2.48–3.87)	257 (205–321)	3.13 (2.48–3.87)	−0.16% (−0.22—0.07)	−0.03% (−0.03—0.02)
Burkina Faso	125 (77–187)	1.55 (1.06–2.22)	314 (196–472)	1.57 (1.07–2.23)	1.52% (1.41–1.63)	0.06% (0.05–0.07)
Burundi	4 (3–6)	0.07 (0.05–0.1)	9 (6–12)	0.07 (0.05–0.1)	1.21% (1.08–1.35)	0.% (0–0.01)
Cabo Verde	5 (3–7)	1.52 (1.03–2.14)	9 (6–13)	1.53 (1.04–2.16)	0.81% (0.58–1.14)	0.04% (0.03–0.06)
Cambodia	10 (2–29)	0.09 (0.03–0.24)	17 (4–49)	0.1 (0.03–0.28)	0.77% (0.5–1.4)	0.11% (0.06–0.16)
Cameroon	47 (28–75)	0.53 (0.35–0.77)	147 (86–236)	0.54 (0.35–0.79)	2.09% (1.94–2.26)	0.09% (0.07–0.11)
Canada	17 (13–21)	0.06 (0.05–0.07)	26 (21–32)	0.06 (0.05–0.07)	0.52% (0.39–0.67)	−0.07% (−0.08—0.06)
Central African Republic	43 (28–64)	1.79 (1.24–2.49)	87 (56–128)	1.8 (1.25–2.5)	1.01% (0.92–1.1)	0.02% (0.02–0.03)
Chad	78 (49–114)	1.53 (1.06–2.14)	215 (132–324)	1.55 (1.06–2.15)	1.76% (1.59–1.93)	0.07% (0.05–0.08)
Chile	141 (119–166)	1.14 (0.97–1.33)	292 (254–335)	1.44 (1.25–1.66)	1.07% (0.91–1.24)	0.4% (−0.18–0.99)
China	4975 (1540–11580)	0.41 (0.14–0.89)	6665 (2962–13327)	0.46 (0.19–0.95)	0.34% (−0.06–1.07)	0.57% (0.49–0.65)
Colombia	6 (4–8)	0.02 (0.02–0.03)	11 (8–15)	0.02 (0.02–0.03)	0.84% (0.59–1.16)	0.04% (0.03–0.04)
Comoros	0 (0–0)	0.01 (0.01–0.02)	0 (0–0)	0.01 (0.01–0.02)	0.59% (0.38–0.89)	0.01% (0–0.02)
Congo	14 (8–22)	0.64 (0.42–0.91)	32 (20–48)	0.65 (0.43–0.93)	1.27% (1.07–1.59)	0.05% (0.04–0.06)
Cook Islands	0 (0–0)	0 (0–0)	0 (0–0)	0 (0–0)	0% (0–0)	0% (0–0)
Costa Rica	1 (0–1)	0.02 (0.02–0.03)	1 (1–1)	0.02 (0.02–0.03)	0.95% (0.69–1.3)	0.04% (0.03–0.04)

location	Num_1990	ASR_1990	Num_2019	ASR_2019	Num_change	EAPC_CI
Croatia	121 (101–142)	2.21 (1.85–2.6)	95 (82–110)	1.92 (1.65–2.23)	−0.21% (−0.27—0.14)	−0.42% (−0.47—0.37)
Cuba	2 (1–2)	0.01 (0.01–0.02)	2 (1–3)	0.01 (0.01–0.02)	0.13% (−0.08–0.37)	−0.07% (−0.11—0.04)
Cyprus	2 (1–2)	0.21 (0.17–0.27)	4 (3–5)	0.26 (0.22–0.32)	1.28% (1.07–1.56)	0.44% (0.28–0.61)
Czechia	39 (31–47)	0.34 (0.27–0.42)	42 (33–51)	0.33 (0.26–0.4)	0.07% (−0.02–0.16)	−0.13% (−0.19—0.08)
Democratic People's Republic of Korea	96 (21–261)	0.48 (0.1–1.28)	128 (30–335)	0.48 (0.11–1.24)	0.32% (0.06–0.76)	0.03% (−0.02–0.08)
Democratic Republic of the Congo	589 (374–876)	1.78 (1.24–2.45)	1427 (908–2117)	1.79 (1.24–2.47)	1.42% (1.33–1.53)	0.03% (0.02–0.04)
Denmark	0 (0–0)	0 (0–0)	0 (0–0)	0.01 (0–0.01)	0.98% (0.38–2.23)	0.72% (0.28–1.17)
Djibouti	0 (0–0)	0.07 (0.05–0.1)	1 (1–1)	0.07 (0.05–0.1)	1.52% (1.25–1.87)	0.02% (0.02–0.03)
Dominica	0 (0–0)	0.01 (0.01–0.02)	0 (0–0)	0.01 (0.01–0.02)	−0.05% (−0.16–0.11)	−0.08% (−0.11—0.06)
Dominican Republic	1 (1–2)	0.01 (0.01–0.02)	2 (1–2)	0.01 (0.01–0.02)	0.55% (0.35–0.84)	−0.07% (−0.11—0.04)
Ecuador	0 (0–1)	0 (0–0.01)	1 (0–1)	0 (0–0.01)	1.01% (0.52–1.71)	0.25% (−0.1–0.61)
Egypt	2661 (1433–4416)	5.21 (3–8.45)	5094 (2746–8425)	5.2 (2.94–8.38)	0.91% (0.76–1.12)	−0.03% (−0.04—0.02)
El Salvador	5 (4–6)	0.11 (0.08–0.13)	7 (5–8)	0.11 (0.08–0.14)	0.38% (0.26–0.51)	0.04% (0.04–0.05)
Equatorial Guinea	1 (0–1)	0.16 (0.09–0.24)	2 (1–4)	0.15 (0.09–0.24)	2.71% (2.39–3)	−0.07% (−0.07—0.06)
Eritrea	11 (8–15)	0.39 (0.3–0.5)	26 (20–35)	0.39 (0.3–0.5)	1.35% (1.23–1.46)	0.02% (0.01–0.02)
Estonia	44 (36–53)	2.49 (2.04–3)	39 (32–48)	2.46 (2.03–2.97)	−0.1% (−0.16—0.02)	−0.04% (−0.04—0.03)
Eswatini	0 (0–0)	0 (0–0.01)	0 (0–0)	0 (0–0.01)	0.39% (0.16–0.75)	0.01% (0.01–0.01)
Ethiopia	629 (481–797)	1.29 (1.04–1.61)	1372 (1051–1745)	1.29 (1.03–1.6)	1.18% (1.1–1.26)	−0.01% (−0.01–0)
Fiji	0 (0–0)	0 (0–0.01)	0 (0–0)	0 (0–0.01)	0.15% (−0.08–0.48)	0.05% (0.04–0.06)
Finland	0 (0–0)	0 (0–0.01)	0 (0–0)	0 (0–0.01)	0.17% (−0.2–0.88)	−0.02% (−0.26–0.23)
France	54 (19–116)	0.09 (0.03–0.2)	64 (24–141)	0.09 (0.03–0.2)	0.19% (−0.08–0.5)	0.01% (−0.04–0.07)
Gabon	6 (3–9)	0.63 (0.41–0.91)	11 (7–17)	0.64 (0.41–0.93)	0.94% (0.82–1.13)	0.07% (0.06–0.09)

location	Num_1990	ASR_1990	Num_2019	ASR_2019	Num_change	EAPC_CI
Gambia	4 (3–7)	0.52 (0.35–0.77)	11 (7–18)	0.53 (0.35–0.78)	1.52% (1.42–1.62)	0.09% (0.07–0.1)
Georgia	381 (313–468)	6.52 (5.35–8.01)	210 (176–251)	5.26 (4.42–6.3)	−0.45% (−0.51—0.39)	−1.04% (−1.23–−0.85)
Germany	2 (1–3)	0 (0–0)	4 (2–6)	0 (0–0.01)	0.8% (0.15–2.42)	1.78% (1.28–2.28)
Ghana	203 (131–299)	1.53 (1.06–2.12)	476 (316–696)	1.56 (1.08–2.18)	1.34% (1.18–1.5)	0.07% (0.06–0.08)
Greece	0 (0–1)	0 (0–0.01)	0 (0–0)	0 (0–0)	−0.1% (−0.33–0.2)	−0.43% (−0.68–−0.17)
Greenland	0 (0–0)	0.01 (0.01–0.02)	0 (0–0)	0.01 (0.01–0.02)	0.14% (−0.02–0.37)	−0.09% (−0.1–−0.08)
Grenada	0 (0–0)	0.01 (0.01–0.02)	0 (0–0)	0.01 (0.01–0.02)	0.21% (0.02–0.44)	−0.08% (−0.12–−0.05)
Guam	0 (0–0)	0.01 (0.01–0.02)	0 (0–0)	0.01 (0.01–0.02)	0.25% (0.12–0.43)	0.14% (0.12–0.16)
Guatemala	1 (1–2)	0.02 (0.02–0.03)	3 (2–5)	0.02 (0.02–0.03)	1.46% (1.2–1.72)	0.05% (0.04–0.06)
Guinea	28 (17–44)	0.53 (0.34–0.78)	61 (36–97)	0.54 (0.35–0.8)	1.18% (1.03–1.3)	0.09% (0.07–0.11)
Guinea–Bissau	14 (8–20)	1.55 (1.05–2.16)	28 (18–41)	1.57 (1.07–2.18)	1.05% (0.95–1.15)	0.06% (0.05–0.08)
Guyana	2 (2–3)	0.29 (0.23–0.37)	2 (2–3)	0.29 (0.22–0.36)	0.03% (−0.05–0.15)	−0.07% (−0.09–−0.04)
Haiti	1 (1–1)	0.01 (0.01–0.02)	2 (1–3)	0.01 (0.01–0.02)	0.96% (0.75–1.24)	−0.07% (−0.1–−0.04)
Honduras	1 (1–1)	0.02 (0.02–0.03)	2 (1–3)	0.02 (0.02–0.03)	1.34% (1.08–1.61)	0.03% (0.02–0.04)
Hungary	133 (105–164)	1.15 (0.9–1.43)	130 (102–161)	1.14 (0.89–1.42)	−0.02% (−0.1–0.06)	−0.03% (−0.04–−0.03)
Iceland	0 (0–0)	0 (0–0)	0 (0–0)	0 (0–0)	0.3% (−0.05–0.78)	0% (0–0)
India	4314 (3416–5378)	0.57 (0.46–0.69)	9041 (7379–10973)	0.66 (0.54–0.79)	1.1% (0.96–1.25)	0.69% (0.56–0.82)
Indonesia	499 (119–1524)	0.26 (0.07–0.74)	354 (97–957)	0.14 (0.04–0.36)	−0.29% (−0.45–−0.01)	−3.23% (−3.82–−2.64)
Iran (Islamic Republic of)	3029 (2435–3750)	5.97 (4.94–7.17)	5760 (4753–6977)	6.55 (5.4–7.93)	0.9% (0.69–1.17)	0.19% (0.09–0.3)
Iraq	1972 (1013–3322)	13.05 (7.38–20.85)	5489 (2905–8992)	13.08 (7.38–20.67)	1.78% (1.45–2.12)	−0.03% (−0.04–−0.01)
Ireland	3 (1–7)	0.07 (0.02–0.18)	4 (1–9)	0.07 (0.02–0.18)	0.39% (0.06–1.01)	0.11% (0.09–0.13)
Israel	20 (8–37)	0.41 (0.17–0.77)	39 (18–71)	0.41 (0.17–0.77)	0.97% (0.66–1.33)	0.04% (0.03–0.06)
Italy	559 (466–658)	0.9 (0.75–1.08)	4295 (3667–5032)	5.76 (4.88–6.83)	6.69% (5.84–7.64)	0.94% (−0.65–2.56)

location	Num_1990	ASR_1990	Num_2019	ASR_2019	Num_change	EAPC_CI
Jamaica	0 (0–1)	0.01 (0.01–0.02)	0 (0–1)	0.01 (0.01–0.02)	0.23% (0.07–0.44)	−0.07% (−0.11–−0.04)
Japan	79 (23–239)	0.06 (0.02–0.18)	33 (17–69)	0.03 (0.01–0.06)	−0.59% (−0.73–−0.2)	−3.79% (−4.57–−3.01)
Jordan	182 (144–224)	5.64 (4.63–6.69)	1000 (825–1206)	8.55 (7.11–10.07)	4.5% (4.03–5.14)	2.06% (1.66–2.47)
Kazakhstan	26055 (21264–31968)	164.09 (135.28–197.75)	23986 (19796–28908)	127.56 (105.34–153.8)	−0.08% (−0.14–−0.01)	−1.22% (−1.44–−0.99)
Kenya	254 (192–322)	1.14 (0.91–1.41)	579 (446–736)	1.13 (0.9–1.41)	1.29% (1.2–1.38)	0.03% (−0.02–0.08)
Kiribati	0 (0–0)	0 (0–0.01)	0 (0–0)	0 (0–0.01)	0.63% (0.34–0.99)	0.06% (0.05–0.07)
Kuwait	88 (44–147)	5.01 (2.76–7.92)	251 (132–450)	5.15 (2.87–8.18)	1.86% (1.26–2.52)	0.06% (0.04–0.08)
Kyrgyzstan	2757 (2259–3382)	68.56 (56.57–82.4)	6211 (5183–7653)	95.61 (80.36–116.11)	1.25% (1.08–1.45)	1.30% (0.94–1.67)
Lao People's Democratic Republic	4 (1–14)	0.1 (0.03–0.28)	8 (2–23)	0.11 (0.03–0.31)	0.79% (0.45–1.14)	0.03% (0.02–0.05)
Latvia	63 (51–76)	2.04 (1.67–2.46)	43 (37–50)	1.82 (1.56–2.13)	−0.31% (−0.38–−0.23)	−0.23% (−0.31–−0.16)
Lebanon	398 (214–651)	13.14 (7.32–21.37)	689 (380–1124)	13.21 (7.38–20.88)	0.73% (0.48–0.99)	−0.02% (−0.04–0)
Lesotho	0 (0–0)	0 (0–0.01)	0 (0–0)	0 (0–0.01)	0.14% (−0.07–0.46)	0.02% (0.02–0.03)
Liberia	9 (5–14)	0.53 (0.34–0.78)	25 (14–39)	0.54 (0.35–0.8)	1.72% (1.52–1.91)	0.06% (0.05–0.08)
Libya	484 (245–832)	12.87 (7.24–20.5)	962 (520–1587)	13.1 (7.31–20.88)	0.99% (0.55–1.56)	0% (−0.02–0.03)
Lithuania	115 (96–139)	2.89 (2.41–3.44)	85 (74–98)	2.46 (2.11–2.86)	−0.27% (−0.33–−0.19)	−0.4% (−0.57–−0.22)
Luxembourg	0 (0–0)	0.04 (0.03–0.05)	0 (0–0)	0.04 (0.03–0.05)	0.6% (0.37–0.85)	−0.08% (−0.26–−0.1)
Madagascar	8 (6–12)	0.07 (0.05–0.1)	20 (13–29)	0.07 (0.05–0.1)	1.32% (1.17–1.5)	0.02% (0.01–0.03)
Malawi	36 (26–48)	0.39 (0.3–0.5)	74 (54–99)	0.39 (0.3–0.5)	1.05% (0.95–1.15)	0.01% (0–0.02)
Malaysia	18 (5–54)	0.1 (0.03–0.26)	32 (9–86)	0.1 (0.03–0.27)	0.77% (0.5–1.4)	0.01% (0.01–0.01)
Maldives	0 (0–1)	0.1 (0.03–0.28)	0 (0–1)	0.1 (0.03–0.28)	1.14% (0.45–2.35)	0.01% (−0.01–0.03)
Mali	112 (73–164)	1.54 (1.04–2.12)	298 (187–447)	1.55 (1.04–2.15)	1.65% (1.49–1.78)	0.05% (0.04–0.06)
Malta	0 (0–0)	0 (0–0)	0 (0–0)	0 (0–0)	0.12% (−0.15–0.5)	0.03% (0.02–0.04)
Marshall Islands	0 (0–0)	0 (0–0.01)	0 (0–0)	0 (0–0.01)	0.19% (−0.08–0.58)	0.06% (0.05–0.07)
Mauritania	28 (18–40)	1.52 (1.04–2.13)	59 (38–86)	1.54 (1.04–2.15)	1.13% (1.06–1.21)	0.07% (0.06–0.08)
Mauritius	1 (0–4)	0.1 (0.03–0.3)	1 (0–3)	0.1 (0.03–0.3)	0.1% (−0.15–0.81)	0% (0–0)

location	Num_1990	ASR_1990	Num_2019	ASR_2019	Num_change	EAPC_CI
Mexico	74 (19–234)	0.09 (0.02–0.29)	58 (22–148)	0.05 (0.02–0.12)	−0.22% (−0.45–0.47)	−1.68% (−2.45–−0.91)
Micronesia (Federated States of)	0 (0–0)	0 (0–0.01)	0 (0–0)	0 (0–0.01)	−0.04% (−0.25–0.27)	0.06% (0.05–0.08)
Monaco	0 (0–0)	0 (0–0)	0 (0–0)	0 (0–0)	0% (0–0)	0% (0–0)
Mongolia	1036 (433–2102)	55.98 (25.19–100.22)	1858 (815–3484)	55.52 (25.61–100.56)	0.79% (0.4–1.29)	0% (−0.02–0.01)
Montenegro	7 (6–9)	1.14 (0.9–1.41)	8 (6–10)	1.14 (0.9–1.41)	0.09% (0–0.21)	−0.02% (−0.03–−0.02)
Morocco	3097 (1623–5184)	13.12 (7.19–21.2)	4938 (2672–7969)	13.18 (7.26–21)	0.59% (0.35–0.91)	−0.03% (−0.05–−0.01)
Mozambique	50 (36–65)	0.39 (0.31–0.51)	114 (83–151)	0.39 (0.3–0.5)	1.29% (1.18–1.43)	−0.01% (−0.01–0)
Myanmar	44 (11–133)	0.11 (0.03–0.28)	59 (15–167)	0.11 (0.03–0.3)	0.33% (0.12–0.75)	0.02% (0.01–0.03)
Namibia	0 (0–0)	0 (0–0)	0 (0–0)	0 (0–0)	0.68% (0.43–1.08)	0.06% (0.05–0.07)
Nauru	0 (0–0)	0 (0–0)	0 (0–0)	0 (0–0)	0% (0–0)	0% (0–0)
Nepal	1 (0–1)	0 (0–0.01)	2 (1–2)	0.01 (0–0.01)	0.92% (0.48–1.57)	0.73% (0.58–0.88)
Netherlands	14 (4–30)	0.09 (0.03–0.19)	16 (6–35)	0.09 (0.03–0.2)	0.19% (−0.06–0.55)	0.05% (0–0.1)
New Zealand	1 (1–2)	0.03 (0.02–0.05)	2 (1–3)	0.04 (0.02–0.06)	0.71% (0.34–1.05)	0.12% (−0.12–0.36)
Nicaragua	1 (0–1)	0.02 (0.02–0.03)	1 (1–2)	0.02 (0.02–0.03)	1.02% (0.75–1.35)	0.04% (0.03–0.04)
Niger	102 (63–153)	1.53 (1.05–2.15)	302 (183–464)	1.55 (1.06–2.19)	1.97% (1.83–2.1)	0.06% (0.05–0.07)
Nigeria	419 (248–657)	0.52 (0.34–0.76)	1055 (610–1725)	0.53 (0.35–0.79)	1.52% (1.37–1.68)	0.12% (0.1–0.14)
Niue	0 (0–0)	0 (0–0)	0 (0–0)	0 (0–0)	0% (0–0)	0% (0–0)
North Macedonia	23 (18–29)	1.14 (0.9–1.4)	28 (22–34)	1.13 (0.88–1.39)	0.18% (0.08–0.3)	−0.04% (−0.05–−0.04)
Northern Mariana Islands	0 (0–0)	0.01 (0.01–0.02)	0 (0–0)	0.01 (0.01–0.02)	−0.08% (−0.21–0.15)	0.13% (0.11–0.16)
Norway	9 (6–12)	0.2 (0.13–0.27)	22 (15–30)	0.37 (0.25–0.52)	1.42% (1.18–1.68)	2.42% (1.75–3.11)
Oman	85 (45–145)	4.94 (2.83–7.88)	237 (119–415)	4.9 (2.8–7.67)	1.77% (1.21–2.3)	−0.04% (−0.1–0.01)
Pakistan	1532 (335–4485)	1.75 (0.42–5.49)	3195 (635–9540)	1.74 (0.4–5.58)	1.08% (0.79–1.32)	−0.01% (−0.02–0.01)
Palau	0 (0–0)	0 (0–0)	0 (0–0)	0 (0–0)	0% (0–0)	0% (0–0)
Palestine	226 (117–387)	13.16 (7.3–21.21)	621 (332–1027)	13.11 (7.41–20.54)	1.75% (1.49–2.04)	−0.05% (−0.07–−0.03)
Panama	0 (0–1)	0.02 (0.02–0.03)	1 (1–1)	0.02 (0.02–0.03)	1.03% (0.81–1.29)	0.03% (0.02–0.03)

location	Num_1990	ASR_1990	Num_2019	ASR_2019	Num_change	EAPC_CI
Papua New Guinea	0 (0–0)	0 (0–0.01)	0 (0–1)	0 (0–0.01)	1.41% (0.94–1.95)	0.06% (0.05–0.07)
Paraguay	12 (10–15)	0.38 (0.31–0.46)	24 (20–29)	0.37 (0.31–0.45)	0.96% (0.83–1.12)	−0.01% (−0.01–0)
Peru	6 (5–9)	0.03 (0.03–0.04)	11 (9–14)	0.03 (0.03–0.04)	0.75% (0.57–0.97)	0% (0–0)
Philippines	2 (1–3)	0 (0–0)	3 (1–5)	0 (0–0)	0.71% (0.26–1.28)	0% (0–0)
Poland	401 (328–485)	1 (0.81–1.22)	425 (366–493)	0.93 (0.79–1.09)	0.06% (−0.03–0.17)	−0.1% (−0.15–−0.04)
Portugal	45 (19–82)	0.41 (0.17–0.78)	52 (25–103)	0.41 (0.17–0.78)	0.17% (−0.14–0.56)	0% (−0.01–0.01)
Puerto Rico	11 (8–14)	0.29 (0.23–0.37)	12 (10–15)	0.29 (0.23–0.36)	0.11% (−0.03–0.27)	−0.05% (−0.08–−0.03)
Qatar	8 (6–10)	1.68 (1.35–2.06)	52 (41–66)	1.68 (1.37–2.04)	5.9% (5.28–6.54)	−0.21% (−0.36–−0.06)
Republic of Korea	14 (10–18)	0.03 (0.02–0.04)	18 (14–24)	0.03 (0.02–0.04)	0.35% (0.11–0.66)	−0.02% (−0.03–−0.01)
Republic of Moldova	778 (643–929)	17.07 (14.15–20.34)	768 (631–923)	16.93 (14–20.2)	−0.01% (−0.09–0.06)	−0.02% (−0.02–−0.02)
Romania	768 (640–926)	3.08 (2.54–3.71)	859 (737–985)	3.96 (3.42–4.6)	0.12% (0–0.25)	0.9% (0.65–1.16)
Russian Federation	17908 (14797–21262)	10.79 (8.98–12.76)	19163 (15764–22876)	10.89 (9.12–12.87)	0.07% (0.01–0.13)	0.05% (0.03–0.06)
Rwanda	5 (3–7)	0.07 (0.05–0.1)	9 (6–14)	0.07 (0.05–0.1)	0.87% (0.73–1.02)	−0.01% (−0.02–0)
Saint Kitts and Nevis	0 (0–0)	0 (0–0)	0 (0–0)	0 (0–0)	0% (0–0)	0% (0–0)
Saint Lucia	0 (0–0)	0.01 (0.01–0.02)	0 (0–0)	0.01 (0.01–0.02)	0.37% (0.12–0.74)	−0.07% (−0.1–−0.05)
Saint Vincent and the Grenadines	0 (0–0)	0.01 (0.01–0.02)	0 (0–0)	0.01 (0.01–0.02)	0.08% (−0.12–0.35)	−0.08% (−0.11–−0.05)
Samoa	0 (0–0)	0 (0–0.01)	0 (0–0)	0 (0–0.01)	0.28% (0.04–0.59)	0.06% (0.05–0.07)
San Marino	0 (0–0)	0 (0–0)	0 (0–0)	0 (0–0)	0% (0–0)	0% (0–0)
Sao Tome and Principe	0 (0–0)	0.12 (0.07–0.19)	0 (0–0)	0.12 (0.07–0.19)	0.88% (0.75–1.1)	0.1% (0.08–0.12)
Saudi Arabia	725 (370–1237)	4.99 (2.8–7.96)	1993 (1026–3486)	5.04 (2.85–7.98)	1.75% (1.13–2.47)	0.01% (−0.01–0.03)
Senegal	34 (20–55)	0.52 (0.35–0.76)	76 (45–121)	0.53 (0.35–0.79)	1.21% (1.07–1.36)	0.09% (0.07–0.11)
Serbia	226 (188–268)	2.22 (1.84–2.66)	248 (208–287)	2.42 (2.04–2.81)	0.1% (0–0.23)	0.34% (0.23–0.44)
Seychelles	0 (0–0)	0.1 (0.03–0.29)	0 (0–0)	0.1 (0.03–0.29)	0.28% (0.03–0.84)	0.02% (0.02–0.02)
Sierra Leone	17 (11–27)	0.53 (0.34–0.78)	42 (25–67)	0.54 (0.34–0.8)	1.47% (1.31–1.61)	0.07% (0.05–0.08)
Singapore	0 (0–0)	0.01 (0–0.01)	0 (0–1)	0.01 (0–0.01)	0.9% (0.53–1.39)	0.01% (0–0.01)
Slovakia	44 (36–54)	0.81 (0.65–0.98)	46 (38–54)	0.74 (0.6–0.89)	0.03% (−0.05–0.13)	−0.25% (−0.33–−0.16)

location	Num_1990	ASR_1990	Num_2019	ASR_2019	Num_change	EAPC_CI
Slovenia	29 (24–35)	1.37 (1.11–1.65)	32 (27–37)	1.3 (1.08–1.56)	0.1% (0.01–0.2)	−0.21% (−0.26–−0.15)
Solomon Islands	0 (0–0)	0 (0–0.01)	0 (0–0)	0 (0–0.01)	0.92% (0.56–1.36)	0.06% (0.05–0.07)
Somalia	27 (19–36)	0.39 (0.3–0.49)	77 (56–103)	0.39 (0.3–0.5)	1.88% (1.72–2.08)	0% (0–0.01)
South Africa	19 (14–26)	0.05 (0.04–0.07)	28 (21–36)	0.05 (0.04–0.06)	0.46% (0.36–0.58)	−0.06% (−0.08–−0.05)
South Sudan	71 (53–90)	1.25 (0.99–1.53)	114 (86–144)	1.26 (1.01–1.54)	0.61% (0.53–0.68)	0.04% (0.04–0.04)
Spain	1 (1–2)	0 (0–0)	1 (1–2)	0 (0–0)	0.13% (−0.07–0.36)	−0.32% (−0.68–0.03)
Sri Lanka	18 (4–54)	0.1 (0.03–0.27)	22 (6–57)	0.1 (0.03–0.27)	0.22% (0.02–0.8)	−0.01% (−0.01–−0.01)
Sudan	2288 (1202–3842)	13.1 (7.53–20.76)	5070 (2661–8432)	13.17 (7.47–20.85)	1.22% (1.06–1.41)	−0.03% (−0.06–−0.1)
Suriname	0 (0–0)	0.07 (0.06–0.1)	0 (0–1)	0.07 (0.05–0.09)	0.53% (0.38–0.74)	−0.08% (−0.12–−0.05)
Sweden	23 (18–28)	0.25 (0.2–0.31)	44 (36–54)	0.41 (0.33–0.52)	0.92% (0.79–1.11)	1.37% (0.76–1.98)
Switzerland	7 (5–8)	0.09 (0.07–0.11)	11 (9–13)	0.1 (0.08–0.13)	0.6% (0.47–0.74)	0.41% (−0.13–0.95)
Syrian Arab Republic	1439 (739–2441)	13.03 (7.32–20.82)	2027 (1129–3260)	13.37 (7.45–21.26)	0.41% (0.18–0.76)	0.03% (0–0.06)
Taiwan (Province of China)	1 (0–1)	0 (0–0)	1 (0–1)	0 (0–0)	0.06% (−0.3–0.7)	0.17% (0.13–0.2)
Tajikistan	5535 (2492–10858)	123.34 (60.01–214.46)	10887 (4891–20170)	121.88 (58.57–213.93)	0.97% (0.71–1.33)	−0.03% (−0.04–−0.02)
Thailand	65 (16–201)	0.11 (0.03–0.31)	73 (23–186)	0.11 (0.03–0.3)	0.13% (−0.25–1.1)	−0.01% (−0.02–0)
Timor–Leste	1 (0–2)	0.1 (0.03–0.27)	1 (0–4)	0.1 (0.03–0.27)	0.85% (0.72–1.15)	−0.01% (−0.01–0)
Togo	49 (30–74)	1.54 (1.05–2.15)	115 (74–168)	1.55 (1.06–2.18)	1.36% (1.2–1.6)	0.05% (0.04–0.06)
Tokelau	0 (0–0)	0 (0–0)	0 (0–0)	0 (0–0)	0% (0–0)	0% (0–0)
Tonga	0 (0–0)	0 (0–0.01)	0 (0–0)	0 (0–0.01)	0.04% (−0.16–0.28)	0.06% (0.05–0.07)
Trinidad and Tobago	0 (0–0)	0.01 (0.01–0.02)	0 (0–0)	0.01 (0.01–0.02)	0.24% (0.03–0.53)	−0.09% (−0.12–−0.06)
Tunisia	1041 (560–1737)	13.11 (7.39–21.05)	1606 (913–2605)	13.18 (7.49–20.6)	0.54% (0.23–0.94)	−0.04% (−0.06–−0.01)
Turkey	1906 (1525–2374)	3.36 (2.75–4.06)	3297 (2810–3826)	3.77 (3.22–4.38)	0.73% (0.52–0.98)	0.44% (0.32–0.57)
Turkmenistan	1825 (775–3600)	56.57 (26.03–101.9)	2822 (1231–5170)	55.86 (25.25–99.31)	0.55% (0.21–0.91)	−0.04% (−0.07–−0.01)

location	Num_1990	ASR_1990	Num_2019	ASR_2019	Num_change	EAPC_CI
Tuvalu	0 (0–0)	0 (0–0)	0 (0–0)	0 (0–0)	0% (0–0)	0% (0–0)
Uganda	205 (156–259)	1.25 (1–1.54)	504 (379–646)	1.25 (1–1.53)	1.46% (1.35–1.57)	0.01% (0–0.01)
Ukraine	4125 (3381–4960)	6.84 (5.66–8.25)	3720 (3027–4504)	6.8 (5.66–8.19)	−0.1% (−0.16–−0.03)	−0.01% (−0.02–0)
United Arab Emirates	34 (16–60)	1.87 (1.02–3.05)	189 (95–372)	1.88 (1.02–3.08)	4.59% (3.15–6.48)	−0.02% (−0.05–0.01)
United Kingdom	97 (25–298)	0.16 (0.04–0.46)	35 (14–74)	0.05 (0.02–0.12)	−0.64% (−0.79—0.42)	−2.99% (−4.05–−1.92)
United Republic of Tanzania	146 (108–193)	0.58 (0.46–0.73)	287 (214–371)	0.52 (0.4–0.65)	0.97% (0.85–1.11)	−0.33% (−0.44–−0.21)
United States of America	113 (58–219)	0.04 (0.02–0.08)	92 (52–164)	0.02 (0.01–0.04)	−0.19% (−0.39–0.06)	−2.37% (−2.88–−1.86)
United States Virgin Islands	0 (0–0)	0.29 (0.23–0.37)	0 (0–0)	0.29 (0.23–0.36)	0.09% (−0.05–0.26)	−0.07% (−0.09–−0.04)
Uruguay	18 (15–22)	0.54 (0.44–0.66)	21 (17–25)	0.54 (0.43–0.65)	0.16% (0.09–0.22)	0% (−0.01–0)
Uzbekistan	22460 (9914–43740)	124.35 (57.81–220.68)	41079 (18351–76048)	123.53 (58.65–219.16)	0.83% (0.57–1.23)	−0.03% (−0.05–−0.01)
Vanuatu	0 (0–0)	0 (0–0.01)	0 (0–0)	0 (0–0.01)	0.95% (0.54–1.46)	0.03% (0.02–0.04)
Venezuela (Bolivarian Republic of)	3 (2–5)	0.02 (0.02–0.03)	6 (5–9)	0.02 (0.02–0.03)	0.89% (0.62–1.23)	0.02% (0.01–0.03)
Viet Nam	2 (1–3)	0 (0–0)	3 (1–4)	0 (0–0)	0.36% (−0.01–1.06)	0.25% (0.2–0.29)
Yemen	569 (292–961)	5.21 (2.89–8.1)	1525 (767–2630)	5.26 (2.89–8.42)	1.68% (1.46–1.92)	0.02% (0–0.03)
Zambia	30 (22–41)	0.39 (0.31–0.5)	71 (52–95)	0.39 (0.3–0.5)	1.34% (1.24–1.47)	−0.02% (−0.02–−0.02)
Zimbabwe	0 (0–1)	0 (0–0.01)	1 (0–1)	0 (0–0.01)	0.42% (0.24–0.69)	0% (0–0)

Tropical Medicine and Infectious Disease

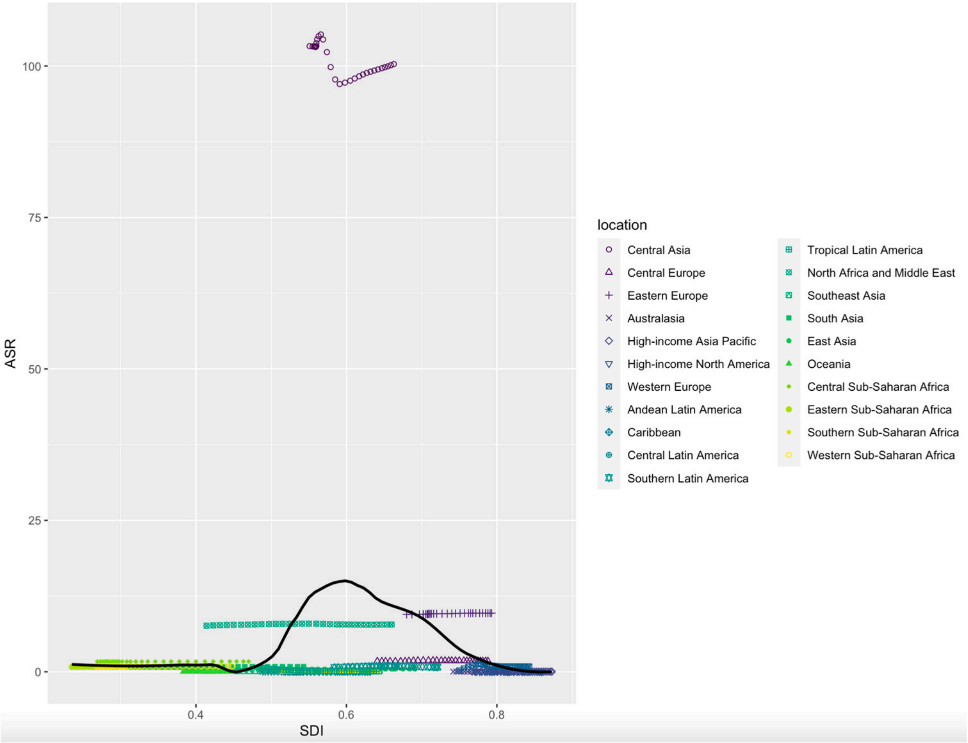

Figure S1. Association between age–standardized incidence of cystic echinococcosis and SDI in 21 GBD regions from 1990 to 2019.

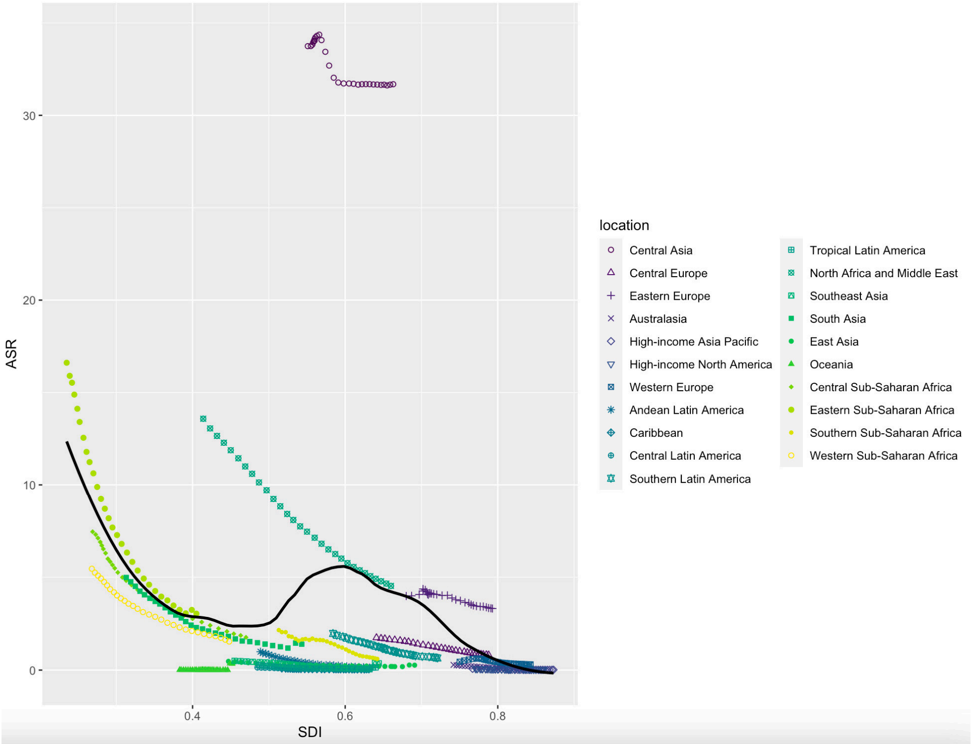

Figure S2. Association between age–standardized DALY rate of cystic echinococcosis and SDI in 21 GBD regions from 1990 to 2019.
